# Advances in Targeted Pesticides with Environmentally Responsive Controlled Release by Nanotechnology

**DOI:** 10.3390/nano8020102

**Published:** 2018-02-11

**Authors:** Bingna Huang, Feifei Chen, Yue Shen, Kun Qian, Yan Wang, Changjiao Sun, Xiang Zhao, Bo Cui, Fei Gao, Zhanghua Zeng, Haixin Cui

**Affiliations:** 1 Institute of Environment and Sustainable Development in Agriculture, Chinese Academic of Agriculture Sciences, Beijing 100081, China; bingnahuang@163.com (B.H.); 18301262687@163.com (F.C.); shenyue@caas.cn (Y.S.); sunchangjiao@caas.cn (C.S.); zhaoxiang@caas.cn (X.Z.); cuibo@caas.cn (B.C.); gaofei@caas.cn (F.G.); zengzhanghua@caas.cn (Z.Z.); 2 College of Plant Protection, Southwest University, Chongqing 400715, China; qiankun1982@163.com

**Keywords:** nanotechnology, precise controlled release, environmental response, nanodelivery system

## Abstract

Pesticides are the basis for defending against major biological disasters and important for ensuring national food security. Biocompatible, biodegradable, intelligent, and responsive materials are currently an emerging area of interest in the field of efficient, safe, and green pesticide formulation. Using nanotechnology to design and prepare targeted pesticides with environmentally responsive controlled release via compound and chemical modifications has also shown great potential in creating novel formulations. In this review, special attention has been paid to intelligent pesticides with precise controlled release modes that can respond to micro-ecological environment changes such as light-sensitivity, thermo-sensitivity, humidity sensitivity, soil pH, and enzyme activity. Moreover, establishing intelligent and controlled pesticide release technologies using nanomaterials are reported. These technologies could increase pesticide-loading, improve the dispersibility and stability of active ingredients, and promote target ability.

## 1. Introduction

Pesticides are indispensable to modern agricultural production. They can effectively reduce plant pests and diseases to improve crop yields. However, traditional pesticide formulations have several disadvantages, such as high organic solvent contents, dust drift, and poor dispersibility, wherein most of the pesticide is lost to the environment and less than 1% remains on the target. This low effectiveness contributes to serious environmental pollution [1,2]. Hence, efforts should be taken to reduce waste, production costs, and environmental pollution associated with pesticides while also extending the duration of pesticide activity on crops.

Problems with traditional pesticide formulations have motivated a new research direction in precise controlled release in recent years. Controlled pesticide release systems are used on farmlands by selecting a suitable and timely administration route to precisely regulate the target pest. This approach aims to minimize crops’ demand for pesticides to gradually achieve more effective, safe pesticide usage via intelligent design that slows and controls pesticide release. To date, studies on the controlled release of pesticides show the system only allows for a simple, slow release rather than a responsive release based on environmental conditions. Thus, if we can achieve precise pesticide release according to environmental light, temperature, soil pH, humidity, and enzyme changes, it may be possible to greatly improve the use of pesticides by reducing waste and pollution. In the past, some pesticides such as pyraclostrobin have been shown to exert a positive effect on crops affected by rice blast fungus. This chemical can induce physiological changes in crops and improve their resistance to the disease, increasing yields considerably [3]. However, this pesticide is highly toxic to aquatic organisms, which has limited its rice crop application [4]. Seltima, a product of BASF, was introduced in 2016 to address pyraclostrobin’s toxicity. Seltima’s microcapsule preparation permits slow release; it is released in a directed manner onto rice leaves based on the pesticide’s humidity sensitivity. Thus, pyraclostrobin can be safely applied to rice and other crops without harming aquatic organisms. The introduction of this product has led to further studies on environmentally responsive pesticide formulations. For example, the temperature-responsive pyraclostrobin microcapsules prepared by Wang et al. offer strong temperature response characteristics and controlled-release functions [5]; Wang et al. embedded abamectin in hollow porous SiO_2_ nanospheres, which improved the pesticide’s action time and stability while controlling its release rate to increase efficiency [6].

Intelligent release in response to environmental agents has a promising future for revolutionizing agricultural production.Guan et al. used UV light as a light source to compare the effects of different microencapsulation factors on pyraclostrobin’s light stability in water. Results showed that microencapsulation reduced the pesticide’s photolytic rate in aquatic environments. Its photolytic rate and initial concentration were negatively correlated [4]. These results provide the basis for the formulation development and rational use of pyraclostrobin. West et al. embedded gold nanoparticles in the PNIPAM microcapsule wall. Under the illumination of near-infrared light, the permeability of microcapsules increases with an increase in temperature [7]. Li et al. synthesized hollow nanoparticles of silica-coated CaCO_3_ as avermectin carriers. The drug’s loading capacity reached 63.6%, effectively preventing avermectin UV light degradation and demonstrating good sustained-release properties [8]. Li et al. prepared pendimethalin polyurethane microcapsules via interfacial polymerization. Results indicated that the average microcapsule particle size was 4.75 μm. The cumulative release of the sample after drying for four hours at 20 °C was 96.57%, while that of samples dried at 130 °C was only 43.56% [9]. Hedaoo et al. prepared pendimethalin microcapsules by interfacial polymerization using polyurethane-amine dendrimer for the wall material. The results indicated that the microcapsules had the lowest cumulative release rate under neutral conditions, whereas the cumulative release rate under acidic conditions was higher than under alkaline conditions [10]. Guo et al. prepared new, functionalized emamectin benzoate microcapsules cross-linked by silica and carboxymethylcellulose. Results showed that the microcapsules could effectively improve emamectin benzoate’s thermal and light stability. Silica-epichlorohydrin-carboxymethylcellulose microcapsules demonstrated a sound biostimulation response to cellulase and pH. The release rate was 28.66% at 1 h with the enzyme addition and reached the maximum at 20 h, while the release rate was no greater than 10% at 30 h with no enzyme addition. The release rate of microcapsules under neutral conditions was lowest; release under acidic and alkaline conditions increased significantly, with alkaline conditions facilitating faster release than acidic conditions. Meanwhile, the controlled-release kinetics of emamectin benzoate microcapsules showed that the microcapsules’ drug release rate was related to temperature: the higher the temperature, the faster the release [11].

**Figure 1:**
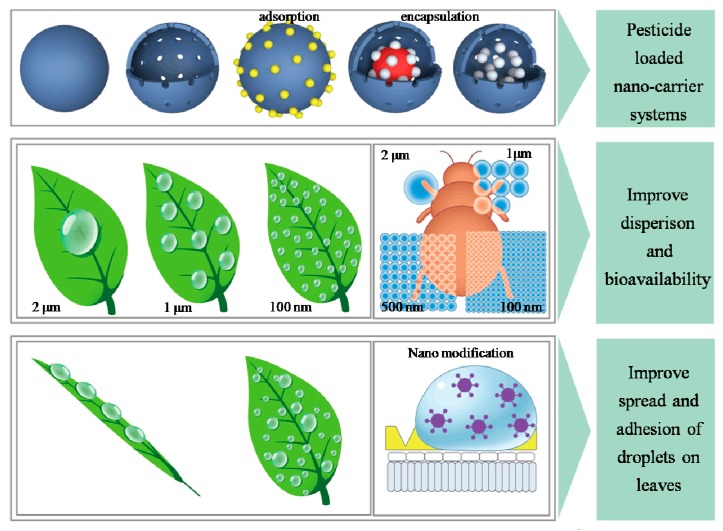
Nano-based pesticide formulation increases bioavailability and efficiency.

The development of nanomaterials and related technologies has provided new ways of creating intelligent nano-pesticides. Nanomaterials have important applications in areas such as pharmacology and biology because their physical and chemical properties are different from those of macroscopic materials. Nanomaterials feature unique surface properties, a small size, and quantum size effects [12]. Nano-microcapsules or nanospheres have been prepared using light-sensitive, thermo-sensitive, humidity-sensitive, and enzyme- and soil pH-sensitive high polymer materials to deliver pesticides. These nanostructured materials are prepared via processes such as adsorption, coupling, encapsulation, and embedding. Such formulations can protect pesticides’ active ingredients and enhance stability, control the release of core materials, reduce or obscure odors, decrease volatility, and isolate components (Figure 1). In recent years, the application of nanomaterials and technologies in the field of pesticides has made considerable strides. In particular, targeted delivery and controlled release of nanomaterials can improve pesticide utilization and reduce residue and pollution. Pesticide nanocapsule formulations have slow release and protection performance and, due to their small size, improvable pesticide droplet ductility, wettability, and target adsorption when spraying fields; these methods provide efficient and environmentally friendly advantages [13,14,15,16]. While progress has been made in research on pesticide nanocapsule formulations, preparation technology warrants further study. Popular pesticide microcapsule preparation methods are shown in Table 1.

Nanostructured materials can also respond to external stimuli such as temperature, light, humidity, and pH to achieve an environmental response and controlled release of pesticides. Therefore, intelligent microcapsules that can respond to the external environment have attracted considerable attention. Although environmentally-responsive nano-drugs have been rapidly developed and applied in the medical field, the application of these materials to pesticides has only recently begun. This paper comprehensively summarizes progress in the applications of polymer materials sensitive to light, temperature, humidity, soil pH, enzymes, and other factors to enhance pesticide release. The environmental response mechanisms leading to targeted release are also discussed.

## 2. Delivery Systems for Environmentally Responsive, Precisely Controlled Pesticide Release

### 2.1. Characteristics and Principles of Light-Sensitive Responsive Polymers and Research Trends in Pesticide Formulation

Given the poor effectiveness and durability of traditional drugs and preparations, researchers have examined various release systems for drugs, including slow release, controlled release, and precision targeting. Timely and appropriate treatment is given to patients by selecting an appropriate drug administration route under controllable external stimulation. In particular, light can precisely control the time and position of a stimulus. Light stimulation can also be delivered from outside the system and used for in vitro control of an irradiated site without requiring changes to the local chemical environment. In addition, many parameters such as light intensity, wavelength, and direction can regulate the reaction process [29]. Medical applications of light-sensitive reactive polymers can be divided into two categories: light-sensitive carrier materials used to deliver drugs, for which the drug release time and site can be precisely controlled by light; and fluorescent substances with an affinity for tumor cells, used as photosensitive reagents. These reagents help to determine the location of a tumor, and light-sensitization reactions destroy tumor cells through photodynamic therapy [30]. For example, gold nanoparticles added to a microcapsule wall can be used to prepare hybrid microcapsules. The microcapsules can be heated by the interaction of gold nanoparticles with a short laser pulse, which causes the microcapsules to fracture. Fluorescent-labeled dextran has been successfully released through this method [31,32]. The use of light-sensitive reactive polymers has advantages including a lack of physical contact and space-time resolution along with characteristics such as convenience, intelligence, and high efficiency. As a result, photo-responsive polymers show great promise in targeted and controlled medication release and tumor cell labeling and tracking.

Photo-responsive polymers exhibit unique physical changes or chemical reactions under light [33]. Relatedly, photo-reactive carrier materials are composed of light-sensitive polymers that undergo rapid physical or chemical changes when exposed to light. After the absorption of light energy, by leveraging photo-responsive polymers’ light sensitivity, the absorbed energy can induce intra- or intermolecular physical changes (such as solubility, color, and conductivity) or chemical reactions such as polymerization, dimerization, isomerization, and photolysis. This approach is widely used in the design and synthesis of photo-responsive carrier materials by introducing light-sensitive groups into polymers’ main or side chains [11]. Photo-responsive carrier material applications in the biomedical field are presented in Table 2.

**Figure 2:**
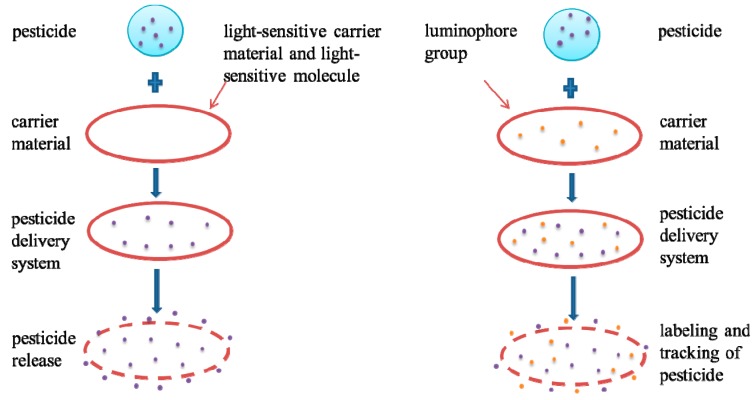
Schematic diagram of light-sensitive polymer response mechanism.

The response mechanisms of light-sensitive responsive polymers include the following: (1) luminophore groups can be added to carrier materials; with light exposure, the luminophore group’s physical and chemical properties change and eventually lead to a change in polymer properties; (2) Light-sensitive carrier materials can be decomposed into ions when exposed to light, thus achieving controlled drug release; (3) Some light-sensitive molecules can convert light into heat energy, which can make the polymers sensitive to light by changing the carrier materials’ internal temperature, as shown in Figure 2.

Many studies on light-sensitive drug release have been performed in the medical field; however, investigations into its applications in pesticides remain limited. The effectiveness of some pesticide formulations is short-lived due to issues including rapid release and light-induced decomposition, despite having positive effects on crops. Hence, frequent administration is needed to ensure efficacy, which limits such pesticides’ applications. To mitigate these issues, researchers have introduced principles from studies of controlled-release drugs into their work on pesticides. Research on light-sensitive responsive pesticides has become a topic of particular interest.

Atta et al. prepared a photo-responsive 2,4-D nano-controlled-release formulation based on light-sensitive organic fluorescent nanoparticles. Their results showed that this controlled-release formulation could be effectively transmitted in plant bodies, which improved herbicidal activity and provided strong fluorescence, cell uptake, and light responsiveness [39]. Atta et al. also prepared a photoresponsive 2,4-D nano-controlled-release formulation based on a coumarin copolymer. This controlled-release formulation showed strong fluorescence and light responsiveness, again effectively transmitting the pesticide into the plant body to improve herbicidal activity. Compared with original pesticides, controlled-release formulations exhibit better thermal stability and lower dissolvability [40].

Photopolymers have been widely applied in material functionalization and intelligent molding owing to their photochemical reaction characteristics. Research has expanded from traditional industrial applications to high-tech fields such as electronics, communications, optical instruments, and medical materials. Light has unique advantages over other stimulation methods. The purity and controllability of photochemical processes also highlight light-responsive polymers’ wide-ranging application prospects. These features are important for agricultural developments: the use of photopolymers in agriculture could enable the precise release of pesticides in the form of nano-microcapsules. We should consider structural stability and potential for environmental pollution when selecting appropriate wall materials and preparation methods for preparing light-sensitive responsive nano-microcapsules that offer high encapsulation efficiency, good drug loading, and precision-controlled pesticide release.

### 2.2. Characteristics, Principles, and Research Trends in Temperature-Responsive Pesticide Formulations

Temperature-sensitive polymers are environmentally responsive materials that have been primarily studied in temperature ranges present in nature and easily achieved by humans. These systems are simple to control and can be applied in vitro and in vivo [11]. A temperature-responsive polymer undergoes property changes as the temperature shifts, which allows for the polymer’s intelligent and reversible response to temperature change. In response to an external temperature shift, temperature-responsive polymers undergo changes in their corresponding physical structure and chemical properties. Materials of temperature control systems include gels, liposomes, polymers, and nanoparticles, as shown in Table 3 [41].

A temperature-responsive polymer’s most representative property is its critical solution temperature. When phase separation of a homogeneous polymer begins to occur at a high temperature, the minimum temperature of the phase separation curve on the concentration-temperature plot is considered to be the lower critical solution temperature (LCST). This characteristic is also known as the upper critical solution temperature [43]. Among such materials, poly-*N*-isopropyl acrylamide (PNIPAM) is a widely used thermosensitive polymer.

**Figure 3:**
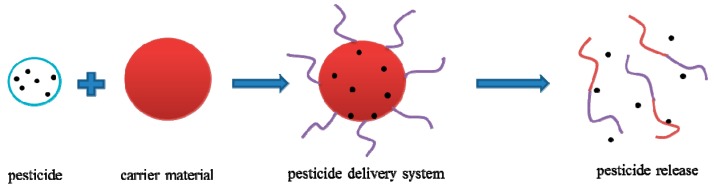
Response mechanism diagram of thermosensitive polymer.

An aqueous PNIPAM solution is thermosensitive. When the temperature is equal to or higher than its LCST (approximately 32 °C), the polymer undergoes phase separation over a wide range of concentrations; when the temperature is lower than the LCST, the precipitated PNIPAM can dissolve rapidly. Cross-linked PNIPAM also has an LCST of approximately 32 °C. Copolymers composed of *N*-isopropyl acrylamide (NIPAM), and certain monomers or polymers and copolymer blends can also have this property. Sensitivity to ambient temperature is of great interest for materials design [44,45] because polymers with an LCST undergo phase separation due to a hydrophobic association between side chains. When the temperature is below the LCST, hydrophilic interactions between water molecules and polymer side chains occur in the presence of strong hydrogen-bonding interactions. The entire polymer becomes water-soluble when the temperature is above the LCST: hydrophobicity occurs because the polymer side chains’ hydrophobic interactions are enhanced, making the entire polymer amphiphilic [12]. When hydrophobic associations between side chains occur, the main chains also change to form a cross-linked network structure. Microscopic phase separation occurs in the polymer side chains, and the polymer viscosity is improved, changing from a stretched irregular coil structure into a tightly colloidal structure [46]. This mechanism is illustrated in Figure 3.

For example, tadpole-shaped (*c*-PNIPAM)-*b*-PCL (annular poly *N*-isopropyl acrylamide-*b*-polycaprolactone block copolymers) synthesized by the Liu group can self-assemble to form PCL-cored water-soluble micelles, which can be used as nanopharmaceutical carriers to achieve controlled drug release at elevated temperatures [47]. McCormick et al. used a room-temperature reversible addition-fragmentation chain transfer polymerization method to synthesize a thermosensitive copolymer composed of NIPAM and dimethylacrylamide [48]. This material was found to be a temperature-sensitive polymer, which could undergo reversible micellization that induced changes in the system’s humidity.

Biomedical applications of thermo-sensitive polymers focus on drug fixation and controlled release. For instance, in cancer treatment, the body’s normal temperature is 37 °C, and the temperature of tumor cells is 40–42 °C. A temperature-sensitive polymer can release drugs based on this temperature difference to control the proliferation of tumor cells [49]. The development of temperature-responsive polymers raises questions about their potential applications in agriculture for addressing problems related to agricultural production. 

Using the copolymer of NIPAM and butyl acrylate as a wall material, Wang et al. prepared temperature-responsive pyraclostrobin microcapsules using an emulsion polymerization method [5]. Their results showed that the microcapsules had temperature responsive properties and a lower critical solution temperature (LCST) of 28.2 °C; the active ingredient could be released quickly when the ambient temperature was higher than 28.2 °C, and the release behavior was inhibited at lower temperatures. This controlled-release property is expected to have applications in rice farming. The group indicated that the acute toxicity LC50 (96 h) value of pyraclostrobin microcapsules was 46.4 mg/L for zebrafish, over 90 times higher than that of original pyraclostrobin; thus, this preparation markedly improves pyraclostrobin’s safety. Binbin et al. used a miniemulsion copolymerization system of NIPAM and acrylic acid (AA) monomers introduced into small molecular droplets of *N*-octane as a template to prepare temperature/pH dual-responsive P(NIPAM-co-AA) nanocapsules [50]. Their results showed that these nanocapsules had good pH sensitivity and temperature response properties.

At present, research on temperature-responsive pesticide formulations is in its nascent stage based on pesticide microcapsules’ carrier materials, critical temperatures, and preparation methods. However, it is important to accelerate the response speed and establish a precise controlled-release model for pesticides at different temperatures as well as to perform field tests. It is also necessary to compare the differences in controlled-release agents’ biological activity under natural and indoor conditions; thus, further study is warranted to examine agents’ mechanisms and pathways to verify their safety in farmland applications.

### 2.3. Characteristics, Principles, and Research Trends in pH-Responsive Pesticide Formulations

pH-responsive polymers comprise the most widely studied class of environmentally responsive polymers, a testament to their solubility, volume, and permeability [50]. pH-responsive polymer materials typically contain weakly acidic or weakly alkaline groups [51], and the molecular segments of these polymers contain a large number of acidic or alkaline groups, such as carboxyl or amine groups. These groups are susceptible to protonation or deprotonation and can capture or release protons through a change in the external pH value [1]. Common pH-responsive polymers include polyacrylic acid (PAA), polyvinylpyridine (PVPY), polymethacrylic acid (PMAA), polymethyl methacrylate-*N*,*N*-dimethylaminoethylbenzene (PDMAEMA), polypropylamine hydrochloride (PAH), and polymethylacrylic acid-2-(diethylamino) ethyl methacrylate (PDEA).

The response principle states that when pH changes, the ionization capacity of a pH-responsive polymer changes accordingly. When the ionization capacity reaches the polymer’s isoelectric point, its solubility changes. pH-responsive polymers are generally classified into two groups: polyacids and polybases [52,53]. The ionization/deionization of polyacids generally occurs within a pH range of 4–8, for which protons are accepted at a low pH and released at a neutral or higher pH, such as PAA and PMAA. Polybases’ pH-responsive polymers typically contain amino groups on their side chains that accept protons under acidic conditions as cations and then release them under alkaline conditions to reduce their solubility, such as in methacrylic acid and *N*,*N*-dimethylaminoethyl methacrylate (DMAEMA, pKb 7.4).

Rapid developments in nanotechnology have created a range of opportunities for intelligent responsive nano-pesticide creation. pH-responsive polymers are used as pesticide carriers because of their high purity, easily controlled properties, simple chemical modification, and good selectivity [11]. These materials are widely used in fields such as pharmaceuticals, cosmetics, food, textiles, and agriculture. However, the application of these materials in targeted and controlled pesticide release is still in an early stage, necessitating in-depth, systematic research and development. Materials used for pH-responsive controlled-release carriers are classified in Table 4.

Sukhorukov et al. first discovered that the permeability of a microcapsule wall composed of a strong polyelectrolyte sodium polystyrene sulfonate and a weak polyelectrolyte is regulated by pH. In recent years, many studies have examined the pH response of microcapsules composed of weak polyelectrolytes. For example, Chang et al. prepared one-component cross-linked polyetherimide (PEI)/PAA hollow microcapsules with a particle template and crosslinking agents [54]. These microcapsules remained stable under extreme environmental conditions: they showed no change in size, and their permeability was pH-sensitive. Imoto et al. reported a preparation method and characterization of pH-responsive nanocapsules [55]. These nanocapsules featured biodegradable polyacrylamide chitosan. The polyglutamic acid groups were responsive to acidic pH conditions, releasing the nanocapsules’ contents; however, release was limited under neutral and alkaline conditions.

pH responsiveness is not only widely used in microcapsule preparations but also in the controlled release of nano-micelles and hydrogels. For example, nano-micelles, developed by Xin et al., include ingredients such as poly-glycol-poly (aspartic acid-cysteine)-poly (aspartic acid-diisopropylethylenediamine) [56]. These materials are sensitive to changes in pH. The disulfide bond in an intermediate crosslinking layer is sensitive to reducing agents and has characteristics useful for intelligent drug release.

Last year, Gu et al. published a summary outlining the latest advances in intelligent biomaterials as well as their design principles, challenges, future directions, and clinical applications. This study included an examination of pH response [57]. In addition to pesticide release applications, the pH gradient in the physiological environment is used to achieve drug release. At the organ level, the pH gradient along the gastrointestinal tract enables organ-specific release of oral medications. In a recent study, a dendrimer conjugate prodrug was released within a tumor due to the breakage of an amide bond in the weakly acidic tumor environment. Under the action of the catalyst azobisisobutyronitrile and the crosslinking agent methacrylamide, the pH-sensitive organic-inorganic copolymer hydrogel P(MAPOSS-co-AA) can be prepared using a radical solution polymerization method. A study of its drug release performance was performed, and results showed that as the pH value increased, the hydrogel’s water absorption was enhanced and the theophylline release rate was reduced owing to system swelling. Thus, stomach stimulation was avoided, and controlled drug release at a fixed point was achieved.

High-DS carboxymethyl chitosan CMCH pharmaceutical carrier particles were prepared by Zeng et al. [58] using an oven drying method, and the particles demonstrated pH sensitivity. CMCH is biocompatible and biodegradable. As such, it is suitable for colonic-specific drug release in the treatment of ulcerative colitis, Crohn’s, and inflammatory bowel diseases.

**Figure 4:**
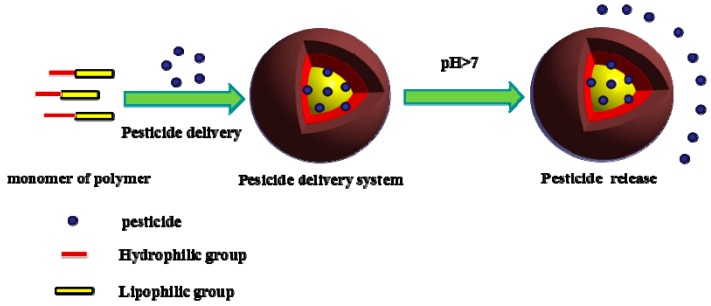
Diagram illustrating the response mechanism of pH-sensitive polymers (pH in controlled-release pesticides under alkaline conditions in alkaline intestinal insects [Lepidoptera]).

To date, an array of medical studies has been conducted on pH response; however, studies regarding pesticides continue to require further development. For example, a PAA/chlorpyrifos/aminated mesoporous silicone sustained-release system was prepared by Lin et al. based on a surface modification method, and it presented a strong pH response [59]. At pH ≤ 7, the pesticide release rate increased as the pH decreased, and the release rate in alkaline conditions was slightly greater than in a neutral environment. PAA/chlorpyrifos/aminated mesoporous silicon also exhibited a strong pH response during release: it was easily released under acidic conditions and demonstrated the best slow-release effects under neutral conditions. Thus, pesticide release can be effectively regulated via pH. In a study conducted by Zhao et al., Schiff base organic molecules were used to control drug release [60]. These chemicals are slightly more stable under alkaline conditions than under acidic conditions; hence, the drug release rate is greater under acidic conditions. The pH-responsive release system under alkaline conditions is illustrated in Figure 4 for an insect (Lepidoptera) target.

pH-responsive polymers have a certain recognition and response to the external environment’s pH value and thus show promise for applications in many fields. Extant studies of pH responsiveness have mainly focused on responsive microcapsules and drug release systems such as hydrogels. Studies on pH-responsive pesticide formulations tend to pertain to precisely targeted and controlled pesticide release, which is beneficial for reducing pesticide drift and improving effective pesticide utilization. These features should, therefore, have considerable advantages in reducing pesticide residues and environmental pollution.

### 2.4. Characteristics, Principles, and Research Trends in Humidity-Sensitive Pesticide Formulations

Humidity-sensitive materials represent a class of materials that possess certain properties, such as material resistance, dielectric constant, and volume; these properties vary with local environmental humidity changes [61]. In recent years, nanoscience and nanomaterials have developed rapidly. Compared with traditional materials, these materials offer advantages such as large specific surface areas, high sensitivity to external environment changes, rapid response, small size, being lightweight, and allowing for easy integration; these qualities provide new directions for the development of humidity-sensitive materials [62]. Humidity-sensitive materials in current use include electrolytes, polymer compounds, ceramic base materials, porous metal oxides, and semiconductor materials [63,64,65]. Among these, electrolytes, polymer compounds, and porous metal oxides are often used in pharmaceutic preparations. Main categories are listed in Table 5.

A polymer humidity-sensitive material contains polar groups (i.e., hydrophilic groups), which interact with water molecules to form hydrogen bonds or through van der Waals force to achieve hygroscopicity [66,67]. Recently, polymer materials have been used as humidity-sensitive materials for humidity sensors; this application has drawn attention to the effectiveness of these systems and their other prospective applications [61].

**Figure 5:**
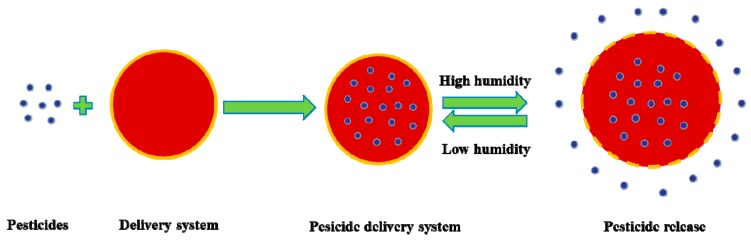
Response mechanism diagram of humidity-sensitive polymer.

Humidity-sensitive materials have been widely applied in the field of sensing technologies. Unlike pH-, temperature-, and light-sensitive materials, these materials have received comparatively less attention to their biological and medical applications. Relative humidity is an important factor that affects microcapsules’ storage stability. Studies conducted by Zeng et al. found that lutein and its microcapsule products show good storage stability under low-humidity conditions [68]; however, their retention rates are significantly reduced when the relative humidity reaches a certain level. In studies conducted by Cui et al., the stability of microcapsule products was also affected by the relative environmental humidity, which is mainly reflected by microcapsule wall materials’ moisture absorption [69]. Thus, a preliminary study was conducted on the wet stability of microcapsule agent products, and results showed that microcapsule products should be stored in relatively low-humidity conditions. According to experimental results, the retention rate of lactic acid microcapsule products decreases as relative humidity increases. Specifically, greater relative environmental humidity decreases the microcapsules’ lifetime. When an environment’s relative humidity changes, microcapsule products absorb moisture and swell. The drugs encapsulated in the microcapsules are then released, as depicted in Figure 5.

In addition, field applications of bacillus thuringiensis preparations present disadvantages such as short residue lifetime; unstable release control; and susceptibility to light, temperature, humidity, and other external conditions [70]. Shan et al.’s microencapsulated Bacillus thuringiensis can reduce spore powder consumption, improve core materials’ stability, negate the effect of UV light [71,72], and reduce environmental humidity and pesticide contact. The released pesticide features extended insecticidal activity, thereby reducing the amount required, minimizing environmental pollution and pesticide damage, and creating an environmentally friendly formulation. 

In recent years, following the development of Seltima by BASF, humidity-sensitive microcapsule pesticide formulations have drawn increasing scholarly attention. The active ingredients in the BASF Seltima microcapsule suspending agents are resistant to release when in contact with water, but they gradually break down over time on crop leaves to produce a pesticide effect. Seltima can be widely used to control rice diseases and has excellent control over rice blast. The unique microcapsule technology of Seltima ensures that active ingredients are precisely released on the rice leaf surface. When droplets dry on the leaf surface, the pyraclostrobin encapsulated in the capsule is quickly released to produce the best control effect; meanwhile, the few capsules that drop into the water of a paddy field remain intact and sink into the sediment, where the active ingredients are degraded by microorganisms. In this way the effects of the pesticide on the aquatic environment are limited. Compared with traditional formulations, this new microcapsule technology features improved toxicological properties and is more environmentally friendly.

At present, research on humidity-sensitive materials for humidity sensors is relatively well established; however, there has been little study on pesticide preparations and pesticides’ humidity sensitivity. Hence, these aspects require further exploration to improve the effective use of pesticide formulations and reduce environmental pollution.

### 2.5. Characteristics, Principles, and Research Trends in Enzyme-Responsive Pesticide Formulations

When a pest interacts with a plant, a series of changes occurs, including changes to the plant enzymes. Enzyme-responsive polymers can physically and chemically react to such stimulation. For example, Yi et al. encapsulated a target protein in nanocapsules with an interfacial polymerization method, which was cross-linked with a polypeptide that can be hydrolyzed by protease. The internal proteins were released by proteolysis [73].

Enzyme-responsive materials are biocompatible and degradable. Material properties can be controlled by enzymatic action, which may result in rearrangement of the materials as well as expansion/contractionand changes in chemical functionalities on the materials’ surface. Changes in the molecular structure may cause changes in the self-assembled morphology, such as the conversion of disordered or spherical micelles into nanofiber structures or the formation of short rods from nanofibers. Changes can also occur to the macro phases, such as hydrogel formation or destruction [74]. If pesticides and environmental response carrier materials (enzymes) are combined to prepare environmentally responsive, controlled-release pesticide agents, the enzyme response could be used to target pests. Thus, enzyme-responsive controlled-release pesticide agents share characteristics with controlled-release agents [11]. Enzyme-responsive materials can be divided into enzyme-responsive polymers, nanoparticles, and hydrogels, as listed in Table 6.

**Figure 6:**
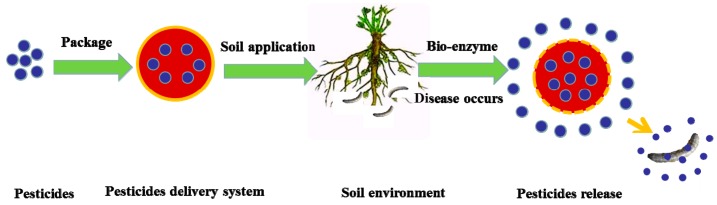
Response mechanism diagram of an enzyme-sensitive polymer.

The controlled-release principle of enzyme-responsive pesticide formulations can be divided into two aspects: the formation of active pesticide molecules via the conversion and degradation of a propesticide; and targeted application, accomplished by the degradation of the encapsulation materials containing the pesticide molecules. When Lepidoptera insects endanger crops, they come into contact with the pesticide microcapsules, and the capsule materials break under the action of cutinase and cell-degrading enzymes to release pesticides, as displayed in Figure 6.

Enzyme-responsive polymers are widely used in anticancer drugs’ transport and release systems. For example, Flelin protease is a preprotein invertase that plays an important role in tumor growth, metastasis, and angiogenesis. Furin-degrading peptide cross-linkers are added to the drug transport carrier. In the cell uptake process, these peptides can gradually degrade and release internal protein. Studies conducted by Nasha et al. found that under the action of intracellular esterase, ERP acetophenols are hydrolyzed rapidly, triggering the reaction of phenolic benzyl alcohol that directly forms carboxylate radicals, which then form a neutral polycation pair with amino groups [75]. This reaction shifts the potential of the cationic polymer from positive to negative, causing the polymer to lose its interaction with the charged DNA such that the compound is rapidly dissociated and releases DNA. Compared with quaternary methyl iodide-ammoniated polyethyleneimine (QPEI-M), a charge reversal cannot occur; its transfection efficiency was found to be 100 to 1000 times higher than that of QPEI-BP without a phenolic ester bond.

Environmental response stimulus factors such as pH, enzymes, temperature, humidity, and light stimulate individual responses, but often a combination of stimulating response factors promotes pesticide release. For example, plants suffering from pests often exhibit decreased pH and increased cellulase. In a study conducted by Guo et al., stimulation by cellulase triggered the release of active ingredients from pH-responsive methylamino abamectin benzoate microcapsules to effectively control pests [11].

The combination of stimulating responsive polymers and enzyme molecules can be used to design and build intelligent enzyme catalysts. Such catalysts have been proposed to respond to the environment based on the enzyme’s catalytic performance, which could be applied to emerging areas including controllable biocatalysis, drug delivery, analytical testing, and intelligent equipment development. Such applications would greatly expand the role of enzyme catalysis [76] and represent an area of interest in research on intelligent biological materials.

Research on enzyme-responsive polymers, nanoparticles, and hydrogels has received significant attention. Many studies have been carried out regarding controlled drug release, biocatalysis, optical sensing, imaging, tissue engineering, and diagnostics. However, a problem that has yet to be fully solved is the dynamic design of polymer hydrogel materials under enzymatic action to make their morphology controllable or chemically reversible. These materials have shown reversible behavior under pH and temperature stimulation, and studies have demonstrated enzymes’ ability to trigger morphological changes in hydrogel polymers. However, it is necessary to further examine the true extent of dynamic/reversible mechanisms that can be developed through the addition of inhibitors or removal of enzyme cofactors to encourage drugs to respond to the disease state [74].

## 3. Future Outlook

This paper summarizes the main types of stimulation-responsive polymers, namely temperature-, pH, humidity-, enzyme-, and light-responsive materials. Notable responsive polymers are introduced with a focus on progress in controlled release under different types of stimulation. A discussion is provided regarding the release processes of responsive carrier materials according to different release mechanisms. Future developments in the application of controlled-release materials in pesticides are also forecasted [73].

Currently, although environmentally responsive systems are well developed in medicine, their applications in agriculture remain limited. Although simple slow release has been achieved, materials showing responsive release based on environmental changes have yet to be developed. The pesticide field also requires continued systematic research, development, and improvement in environmentally responsive, targeted, controlled-release pesticides. In particular, effective systems that are responsive to internal bio-stimulation present a great challenge in this field. Pesticide-responsive controlled-release agents effectively improve pesticide utilization and substantially reduce pesticides’ negative effects on agricultural products and the environment. Agents should have the ability to respond intelligently to the external environment and should have a trigger release of active ingredients to effectively control harmful organisms. Microcapsule pesticide formulations are an important application of this technology in pesticide formulation preparation. Compared with conventional pesticide formulations, these systems have advantages such as long lifetimes, reduced environmental effects, and lower toxicity. These features may also improve rain resistance. In recent years, development prospects in areas related to pesticide-controlled release have drawn much attention [77,78]. If a pesticide microcapsule can be properly applied, the pesticide will be released only at the target tissue. This process allows the pesticide to be used more effectively while ensuring that affected plant tissues receive the optimum therapeutic effect, thus reducing environmental pollution. Therefore, the application of microcapsule materials in pesticides and the development of new environmentally intelligent and responsive pesticide formulations represent new directions in pesticide formulation.

## Figures and Tables

**Table 1:** Main pesticide microcapsule preparation methods.

Method	Preparation Process	Example
Interfacial polymerization	Two reactive monomers are dissolved in two different solvents. When one solvent is dispersed in another solvent, the two monomers undergo a polycondensation reaction at the phase interface to form microcapsules [17].	Natural pyrethrin nanocapsules [18]
In situ polymerization	Two or more water-soluble monomers are polymerized to form a water-insoluble polymer and are deposited on the surface of the core material for coating [19].	S-ethyl dipropylthiocarbamate, acetochlor, atrazine, methotrexate
Emulsion polymerization	A solvent-insoluble monomer is dispersed in a solvent to form a uniform emulsion via mechanical agitation, high-speed shearing, and vigorous shaking with a surfactant (an emulsifier). Then, the polymerization reaction is initiated to form the polymer to achieve core material encapsulation [20].	Abamectin nanocapsule suspension [21], natural pyrethrin nanocapsules [22]
Membrane emulsification	The dispersion phase enters the continuous phase through a shirasu porous glass membrane under inert gas pressure, and the continuous phase breaks into droplets on the membrane surface by the shear forces of the SPG membrane and droplets.	Chlorantraniliprole nanocapsules [23], avermectin nanocapsules [24]
Solvent evaporation	The wall material and core material are dispersed in the organic phase, added to the solution immiscible with the wall material, and the wall material is precipitated to form the microcapsule by heating and evaporating the solvent [25].	Spinosad nanocapsules [26]
Nano-precipitation	The interfacial interaction between solvent and non-solvent disperses the polymer and drug from the oil phase into the aqueous phase. This material can quickly wrap the drug and obtain nanocapsules through precipitation [19].	Pyrazole azoxystrobin nanocapsules [27], azoxystrobin microcapsules [8]
Double coacervation	Two oppositely charged water-soluble polymers form a wall around the water-insoluble pesticide active ingredient, which is a spontaneous liquid-to-liquid separation caused by electrostatic interactions [19].	Azoxystrobin microcapsules [28]

**Table 2:** Light-sensitive polymer classifications.

Classification		Characteristics	Examples
Organic nano-drug carrier	Micelles and vesicles	Prepared from amphiphilic polymers; a photo-responsive amphiphilic polymer can be obtained by introducing light-sensitive groups onto the hydrophobic side. After self-assembly and encapsulation of drug micelles or vesicles, controlled drug release can be achieved.	Poly(ethylene oxide-*b*-methacrylic acid) (PEO-PMAA)
	Liposomes	Achieved by stem grafting light-sensitive groups into polymers to construct a liposomal or hydrophobic region in amphiphilic materials [34].	trans-liposomes and Bis-Azo-PC liposomes
	Hydrogels	A photo-responsive crosslinker breaks when exposed to light; then, the capsule structure disintegrates and the drug is released.	Chlorophyllin, dichromate, aromatic azide, diazo compounds, aromatic nitro compounds, organic halogen compounds
Inorganic nano-drug carrier		Inorganic nanoparticles have a well-controlled size and shape with large surface areas. Unique light-, electricity-, and magnetic-sensitive properties enable functions such as bioimaging, targeted delivery, and collaborative drug therapy with the potential for drug delivery inside cells [35].	Mesoporous silica, gold nanomaterials, iron oxide
Light-sensitive group	Spiropyran	Light treatment leads to reversible structural changes in light-sensitive groups, destroys the carrier structure to release drugs, and reassembles the carrier structure [36].	Benzo thiopyran compound of spiro monoaza crown ethers, benzo-crown ether spiropyran, benzo thiopyran compound of monazo sulfide crown ether [36]
	Azobenzene	In light stimulation, cis-trans isomerism can be reversed to elicit changes in material properties such as color, size, shape, polarity, refractive index, and solubility [37].	dendritic polyamide-amine (PAMAM)
	Nitrobenzene	Light induces irreversible fracture of light-sensitive groups and removes light-sensitive compounds to disassemble micelles.	O-nitrobenzyl alcohol, ortho-nitrobenzal
	Coumarin	Unsaturated double bonds in the structure of coumarin compounds form an extended conjugation system. Most compounds show blue or blue-green fluorescence under UV light [38].	Furocoumarins, psoralens, 5-methoxypsoralen (5-MOP),8-methoxypsoralen (8-MOP)

**Table 3:** Classification of thermosensitive polymers.

Classification	Characteristics	Examples
Hydrogels	When temperature rises to a certain value, hydrogels change from a swollen, soft, transparent state to an opaque state.	Poly(*N*-isopropylacrylamide), polyethylene glycol
Liposomes	Can form lipid bimolecular vesicles to entrap drugs with many different polarities in their inner water phase and bimolecular vesicle membrane; have good biocompatibility and can be metabolized normally [34].	Adriamycin liposomes, daunorubicin liposomes, cytarabine liposomes, paclitaxel liposomes, 5-fluorouracil multiphase liposomes
Polymers	Have a certain response to temperature stimulation.	Poly-*N*-isopropyl, acrylamide, polyvinylpyrrolidone, polyvinyl methyl ether, polyoxyethylene ether, hydroxypropylene cellulose [42]
Nanoparticles	Drugs are embedded or dissolved in the nanoparticles or adsorbed/covalently attached to their surfaces; not susceptible to degradation by enzymes within the cell and can effectively maintain drug activity.	Metal and inorganic nanoparticles

**Table 4:** Classification of pH-responsive carrier materials.

Classification	Characteristics	Examples
Microcapsules	Particle size is generally of micro- or nano-scale; divided into a capsule core, capsule wall, and capsule material. Conformation of polyelectrolytes under different pH conditions change, affecting the microcapsule’s diffusion transmittance.	Supramolecular graft polymer, *N*,*N*-dialkylaminoalkyl acrylate polymer
Polymer microspheres	Generally, feature acid/alkali groups that can be ionized or associated on a macromolecule skeleton.	Polyacrylic acid (PAA), polymethacrylic acid (PMAA)
Hydrogels	Respond to changes in environmental conditions and have relatively large structures; achieve transition between tight and swelling states. Generally, they contain acidic or alkaline pendant groups but with a wide-swelling pH range.	Polyethylene glycol-*b*-poly (4-vinylpyridine) (PEG-*b*-P4VP)
Mesoporous materials	Uniform and adjustable pore size, stable skeletal structure, good biocompatibility, sufficiently large specific surface area, and easily functionalized pore volume surface.	SBA-15, mesoporous silica

**Table 5:** Classification of humidity-sensitive materials.

Classification	Characteristics	Examples
Electrolytes	Good humidity-sensitive response characteristics and simple preparation [1], absorbs water and can be diluted; if outflow occurs, humidity-sensing properties are damaged.	Polyacrylic acid (medical and health), polystyrene sulfonate
Polymer compounds	Wide range of materials, wide relative humidity range, low cost, rapid moisture-sensitive response, excellent heat resistance, and rapid humidity response; however, high resistance temperature coefficient, poor reproducibility and interchangeability, and low pollution tolerance.	Polyimide (plastic), hyperbranched quaternary ammonium salt (disinfectant and bactericide)
Porous metal oxides	Low density, high porosity, large specific surface area, and selective permeability to gas.	MeCr_2_O_4_-Bi_2_O_3_ systems, porous α-Fe_2_O_3_ nanospheres

**Table 6:** Classification of enzyme-responsive materials.

Classification	Characteristics	Examples
Enzyme-responsive polymer	Highly specific, high tropism, efficient, mild reaction conditions.	Kasugamycin (agricultural fungicide), emamectin benzoate (insecticide and acaricide)
Nanoparticles	Catalytic efficiency is lower than that of some organic catalysts; high stability and low cost.	Ferritic nanoparticles, gold nanoparticles, fullerene derivatives, reduced graphene-zinc ferrite magnetic composite nanomaterials (rGO-ZnFe_2_O_4_) (nano-enzyme)
Hydrogel (Glucose-hydroxyethyl methacrylate-dimethylaminoethyl methacrylate hydrogel)	Excellent biocompatibility, degradability, and convenient functionality.	RADA16-I (oligopeptide material), polyethylene glycol diacrylate-methacrylic acid, polymethyl cellulose-glycidyl methacrylate gel

## References

[1] Sun C.J., Cui H.X., Wang Y., Zeng Z.H., Zhao X., Cui B. (2016). Studies on Applications of Nanomaterial and Nanotechnology in Agriculture. J. Agric. Sci. Technol..

[2] Reichenberger S., Bach M., Skitschak A., Frede H. (2007). Mitigation Strategies to Reduce Pesticide Inputs into Ground-and Surface Water and Their Effectiveness: A review. Sci. Total Environ..

[3] Han L.L., Bi L.W., Zhao Z.D., Li D.W. (2011). Adavances in Microcapsules Preparation. Biomass Chem. Eng..

[4] Guan L., Zhang P., Wang X.K., Ren Y.P., Guo B.B., Liu F. (2015). Photodegradation of Pyraclostrobin in Water Environment and Microencapsulation Effect on Its Photostability. J. Agro-Environ. Sci..

[5] Wang N., Qi L., Wang Y., Li X.G. (2017). Preparation and Performance of Thermo-sensitive Pyraclostrobin Microcapsules. Chin. J. Pestic. Sci..

[6] Wang B., Wen L.X., Li X.G., Cheng P., Chen J.F. (2007). A Comparison of Different Drug Loading Methods for Porous Hollow Nano-carriers. J. Beijing Univ. Chem. Technol. Nat. Sci. Ed..

[7] Sershen S.R., Westcott S.L., Halas N.J., West J.L. (2000). Temperature-sensitive Polymer Nanoshell Composites for Photothermally Modulated Drug Delivery. J. Biomed. Mater. Res..

[8] Li Z.Z., Xu S.A., Wen L.X., Liu A.Q., Wang Q., Sun H.Y., Yu W., Chen J.F. (2006). Controlled Release of Avermectin from Porous Hollow Silica Nanoparticles: Influence of Shell Thickness on Loading Efficiency, UV-shielding Property and Release. J. Control. Release.

[9] Li B.X., Wang K., Zhang D.X., Liu F. (2013). Study on the Release-Kinetics of Rolyurethane Microcapsule of Pendimethalin. Pestic. Sci. Adm..

[10] Hedaoo R.K., Tatiya P.D., Mahulikar P.P., Gite V.V. (2014). Fabrication of Dendritic 0 G PAMAM-based Novel Polyurea Microcapsules for Encapsulation of Herbicide and Release Rate from Polymer Shell in Different Environment. Des. Monomers Polym..

[11] Guo M.C. (2016). Preparation and Biological Efficacy Evaluation of Stimuli-Responsive Controlled Release Formulation of Pesticide. Ph.D. Thesis.

[12] Zhang F. (2009). Preparation and Characterization of Magnetic and Thermosensitive Polymer Microcontainers. Ph.D. Thesis.

[13] Nair R., Varghese S.H., Nair B.G., Maekawa T., Yoshida Y., Kumar D.S. (2010). Nanoparticulate Material Delivery to Plants. Plant Sci..

[14] Khot L.R., Sankaran S., Maja J.M., Ehsani R., Schuster E.W. (2012). Applications of Nanomaterials in Agricultural Production and Crop Protection: A review. Crop Prot..

[15] Pinto R.C., Neufeld R.J., Ribeiro A.J., Veiga F. (2006). Nanoencapsulation I Methods for Preparation of Drug Loaded Polymeric Nanoparticles. Nanomed. Nanotechnol. Biol. Med..

[16] Tebaldi M.L., Belardi R.M., Montoro S.R. (2016). Polymers with Nano-encapsulated Functional Polymers. Design and Applications of Nanostructured Polymer Blends and Nanocomposite Systems.

[17] Guo Y.Z. (2014). Study on the Preparation and the Release Mechanism of Acetochlor Microcapsules Produced by Interfacial Polymerization Method. Master’s Thesis.

[18] Zhou Y.F., Wu J., Chen J., Nie W.Y. (2007). Applied Research of APEP-type Polymerizable Emulsifier in the Preparation of Pesticide Nanocapsule. J. Anhui Univ..

[19] Wang A.Q., Wang Y., Wang C.X., Cui B., Sun C.J., Zhao X., Zeng Z.H., Yao J.W., Liu G.Q., Cui H.X. (2018). Research progress on nanometer microencapsulation of pesticide. J. Agric. Sci. Technol..

[20] Song Q., Mei X.D., Huang Q.L., Wang Z.Y., Ning J. (2009). Preparation of Abamectin Microcapsules by Means of Emulsion Polymerization and it Bioactivity. Chin. J. Pestic. Sci..

[21] Shang Q., Shang Z.H., Ci S.Y. (2007). HPLC Analysis of Abamectin Nanocapsules Suspension Concentrate. Agrochemicals.

[22] Wu J., Zhou Y.F., Chen J., Nie W.Y., Shi R. (2008). Preparation of Natural Pyrethrum Nanocapsule by Means of Microemulsion Polymerization. Polym. Mater. Sci. Eng..

[23] Liu B., Zhou X., Yang F., Shen H., Wang S.G., Zhang B., Zhi G., Wu D.C. (2013). Fabrication of Uniform Sized Polylactone Microcapsules by Premix Membrane Emulsification for Ultrasound Imaging. Polym. Chem..

[24] Feng J.G., Xu Y., Luo X.R., Yan H., Wu X.M. (2011). Discussion on the Solvent Evaporation Method for Preparation of Microcapsules and the Development of the Pesticides Microcapsules. Chin. J. Pestic. Sci..

[25] Cao M., Guan P., Hu L., Chen X.P. (2009). The Research Progress and Influential Factors on Preparation of Microcapsules by W/O/W Multiple Emulsion-Solvent Evaporation Method. Chem. Bioeng..

[26] Xu L. (2013). Preparation and Characteristic Analysis of Microcapsule Controlled Releasing Agent of Pesticide Chitosan Copolymer. Master’s Thesis.

[27] Zhou X.Q., Cao L.D., Liu Y.J., Li F.M. (2014). Preparation and PerformanceCharacteristics of Azoxystrobin Microcapsules. Chin. J. Pestic. Sci..

[28] Ma C.J., Tao L.H. (2008). Study on the Technology of Preparing Azoxystrobin Microcapsules by Complex Conservation. J. Anhui Agric. Sci..

[29] Wang S.P. (2014). Construction of Photosensitive Nanocarriers and Their Photo-Responsive Characteristic. Master’s Thesis.

[30] Zhang R.R. (2014). Preparation of Photo- and Temperature-Responsive Nano-Drug Delivery for Controlled Release. Master’s Thesis.

[31] Radt B., Smith T.A., Caruso F. (2010). Optically Addressable Nanostructured Capsules. Adv. Mater..

[32] Angelatos A.S., Radt B., Caruso F. (2005). Light Responsive Polyelectrolyte/Gold Nanoparticle Microcapsules. Phys. Chem. B.

[33] Yu Y.Y. (2009). Preparation and Characterization of Multi-Stimuli Responsive Polymer and Microshperes. Master’s Thesis.

[34] Li X.Y., Zeng F., Zhao Y.R., Ju J., Lv W.L. (2014). Advances in the Research and Development of Liposomal Drug Delivery Systems. Chin. J. New Drugs.

[35] Bi H., YU L.L., Song M.M. (2011). Progress of inorganic nanoparticles as targeted drug nanocarriers. Anhui Univ. Nat. Sci. Ed..

[36] Zhang H.X., Meng X., Li P. (2008). Light and Thermal-Stimuli Responsive Materials. Prog. Chem..

[37] Zhao R.Y. (2014). Design Synthesis and Property of Azo-Polymer with Photo-Responsive Function. Ph.D. Thesis.

[38] Huang Y.F. (2015). Application of Anticancer Drugs with Coumarin Structure. Strait Pharm. J..

[39] Singh P.N.D., Atta S., Bera M., Chattopadhyay T., Paul A., Ikbal M., Maiti M.K. (2015). Nanno-pesticide Formulation Based on Fluorescent Organic Photoresponsive Nanoparticles: For Controlled Release of 2,4-D and Real Time Monitoring of Morphological Changes Induced by 2,4-D in Plant Systems. RSC Adv..

[40] Atta S., Paul A., Banerjee R., Bera M., Ikbal M., Dhara D., PradeepSingh N.D. (2015). Photoresponsive Polymers Based on a Coumarin Moiety for the Controlled Release of Pesticide 2,4-D. RSC Adv..

[41] Torchilin V.P. (2014). Multifunctional, Stimuli-Sensitive Nanoparticulate Systems for Drug Delivery. Nat. Rev. Drug Discov..

[42] Li X.R. (2014). Preparation of the Multi-Responsive Polymer Microspheres and Application in Controlled Drug Release. Master’s Thesis.

[43] Otsuka I., Travelet C., Halila S., Fort S., Pignot-Paintrand I., Narumi A., Borsali R. (2012). Themosponsive Self-assemblies of Cyclic and Branched Oligosaccharide-block-poly(*N*-isopropylamide) Diblock Copolymers into Nanoparticles. Biomacromolecule.

[44] Shen Y., Wang Y., Zhao X., Sun C.J., Cui B., Gao F., Zeng Z.H. (2017). Preparation and Physicochemical Charateristics of Thermo-responsive Emamectin Benzoate Microcapsules. Polymers.

[45] Ren Y.R., Huo D.Q., Zhou H., Hou C.J. (2005). Preparation of Thermally Responsive Microcapsule. Guangxi Dev. Chem. Ind..

[46] Wang Y. (2013). Development of Temperature Stimuli-responsive Water-soluble Polymers. Guangzhou Chem. Ind..

[47] Wan X.J., Liu T., Liu S.Y. (2011). Synthesis of Amphiphilic Tadpole-shaped Linear-cyclic Diblock Copolymers via Ring-opening Polymerization Directly Initiating from Cyclic Precursors and Their Application as Drug Nanocarriers. Biomacromolecule.

[48] Convertine A.J., Lokitz B.S., Vasileva Y., Myrick L.J., Scales C.W., Lowe A.B., McCormick C.L. (2006). Direct Synthesis of Thermally Responsive DMA/NIPAM Diblock and DMA/NIPAM/DMA Triblock Copolymers via Aqueous Room Temperature RAFT Polymerization. Macromolecules.

[49] Mura S., Nicolas J., Couvreur P. (2013). Stimuli-responsive Nanocarriers for Drug Delivery. Nat. Mater..

[50] Xu B.B. (2014). Preparation and Characterization of Magnetic/Temperature/pH Responsive Nanocapsules. Master’s Thesis.

[51] Zhang H. (2013). Preparation of pH-Responsive Polymers Coating Bimodal Mesoporous Materials and Its Application in Drug Delievery Ststem. Master’s Thesis.

[52] Yang Y.Q. (2012). Preparation and Structure-Performance Relationship of pH-Sensitive Polymers and Their Self-Assembled Micelle Drug Delivery System. Ph.D. Thesis.

[53] Lee A.S., Gast A.P., Butun V., Steven P.A. (1999). Characterizing the Structure of pH Dependent Polyelectrolyte Block Copolymer Micelles. Macromolecules.

[54] Tong W.Y., Gao C.Y. (2008). Layer-by-Layer Assembled Microcapsules: Fabrication, Stimuli-responsivity, Loading and Release. Chem. J. Chin. Univ..

[55] Imoto T., Kida T., Matsusaki M., Akashi M. (2010). Preparation and Unique pH-responsive Properties of Novel Biodegradable Nanocapsules Composed of Poly(gamma-glutamic acid) and Chitosan as Weak Polyelectrolytes. Macromol. Biosci..

[56] Shuai X.T., Dai J., Lin S.D. (2012). Nano Micelle Capable of Intelligently Releasing Medicine, Preparation Method and Application Thereof. CN Patent.

[57] Yue L., Aimetti A.A., Robert L., Zhen G. (2016). Bioresponsive Materials. Nat. Rev. Mater..

[58] Zeng J. (2015). The Intelligent Drug Controlled Releasing Hydrogel Based on Crosslinked Carboxymethyl Chitin. Master’s Thesis.

[59] Lin Y.S. (2016). Synthesis, Modification and Application in Slow-Release Pesticide of Mesoporous MCM-41. Master’s Thesis.

[60] Zhao X.J., Wu Y., Jiang B.G. (2006). The Investigation of Synthesis and Decomposition of Amino Acid Salicyadehyde Schiff Base. J. Dalian Natl. Univ..

[61] Li Y.H., Huang Y.D., Liu Y.Y. (2003). Humidity Sensing Polymer Materials. Mater. Sci. Technol..

[62] Lv X. (2008). The Design, Preparation, Performance and Application of Polymeric Humidity Sensitive Materials. Ph.D. Thesis.

[63] Radeva E., Bobev K., Spassov L. (1992). Study and Application of Glow Discharge Polymer Layers as Humidity Sensors. Sens. Actuators B.

[64] Sun L.Y. (1996). Development of Foreign Humidity Sensor. J. Transducer Technol..

[65] Yi H., Li Z.Y., Chen D.Z. (1997). Fabrication of Polymer Film Humidity Sensitive Capacitor. Chin. Instrum..

[66] Liu C.J. (1997). General Situations of High Polymer Humidity Sensors and its Developing Direction. Instrum. Tech. Sens..

[67] Klier J., Weichhard C., Leiderer P. (2000). Wetting Behaviour of Solid and Liquid Hydrogen Films. Phys. B.

[68] Zeng Z.P. (2010). The Study on Microcapsule Preparation of Lutein and Its Stability. Master’s Thesis.

[69] Cui Q.B. (2008). Studies on the Microencapsulation and Application Acidulant. Master’s Thesis.

[70] Cunningham J.C. (1988). Baculoviruses: Their Status Compared to Bacillus Thuringiensis as Microbial Insecticides. Outlook Agric..

[71] You H., Zhou X.M., Xu J.W., Li Y.T., Liao S.J. (2009). Microcapsulation Formulation of Bacillus Thuringiensis for Protection against Ultraviolet Radiation Induced Inactivation. J. Huazhong Agric. Univ..

[72] Johnson F.S. (1976). Average Latitudinal Variation in Ultraviolet Radiation at the earth’s Surface. Photochem. Photobiol..

[73] Jiang T., Hu Y.N., Long Y., Song K. (2015). Recent Progress on the Controlled Release of Stimuli-responsive Microcapsule. Imaging Sci. Photochem..

[74] Bai J.K., Zhang Y., Wang J.X. (2016). Progress Intelligent Materials Based on Enzyme Response. Mater. Rev..

[75] Qiu N.S. (2016). Esterase-Responsive Tumor Cell Selective Gene Delivery System for Cancer Gene Therapy. Ph.D. Thesis.

[76] Zhang Y.F., Ge J., Liu Z. (2015). Recent Advancement of Smart Enzyme Catalysts Based on Stimulus-Responsive Polymers. Polym. Bull..

[77] Hua N.Z. (2010). Development and Recent Progress of Pesticide Microencapsulates. Mod. Agrochem..

[78] Zhao X., Cui H.X., Wang Y., Sun C.J., Cui B., Zeng Z.H. (2017). Development Strategies and Prospects of Nano-based Smart Pesticide Formulation. J. Agric. Food Chem..

